# Brain Ultrasonography in Critically Ill Septic Patients: A Scoping Review

**DOI:** 10.3390/jcm13226920

**Published:** 2024-11-17

**Authors:** Giada Cucciolini, Irene Corda, Francesco Forfori, Francesco Corradi

**Affiliations:** Department of Surgical, Medical, Molecular Pathology and Critical Care Medicine, University of Pisa, 56126 Pisa, Italy; i.corda@studenti.unipi.it (I.C.); francesco.forfori@unipi.it (F.F.); francesco.corradi@unipi.it (F.C.)

**Keywords:** brain ultrasound, TCCS, transcranial doppler, sepsis, multimodal monitoring, POCUS

## Abstract

Sepsis-associated encephalopathy (SAE) is linked to high mortality and impaired neurologic outcome. Brain ultrasonography (US) is a non-invasive tool for cerebral monitoring. A scoping review of the literature in three databases was performed to answer if brain perfusion is altered in sepsis, to determine the role of brain US in guiding resuscitation and its ability to predict the outcome. Randomized controlled trials, clinical trials, observational studies, and systematic reviews on adults with sepsis or septic shock in the ICU were included. A total of 625 articles were screened, and 34 included. There were 85% observational studies and 15% systematic reviews with or without meta-analysis. The majority of studies had a small sample size and used different metrics. The studies focused on cerebral blood flow (CBF) alterations reporting variable results (CBF increased, normal, or decreased). The findings showed a variable rate of cerebral autoregulation (CAR) impairment, with higher incidence in the early stages of sepsis and associations with poor neurological outcomes. However, the impact of CAR and CBF alterations on neurological outcomes and mortality was not clear. Very few studies were found on resuscitation. In conclusion, brain US can identify cerebral perfusions alterations and its usage in sepsis is promising. However, the current body of evidence for its usage is poor and lacks standardization.

## 1. Introduction

Sepsis is a global burden affecting millions of people annually worldwide, with significant rates of morbidity and mortality [1]. Sepsis-associated encephalopathy (SAE) is a widespread brain dysfunction that results from an infection located outside of the central nervous system. This condition is a serious acute neurological disorder ranging from delirium to a state of coma [2,3]. It is a common syndrome affecting up to 50–70% of patients with sepsis and impairing the neurologic outcome. Patients with acute brain dysfunction have higher mortality and morbidity, with reduced quality of life and long-term cognitive impairment [4,5,6,7]. Moreover, some patients present with unexplained delayed awakening or persistent coma [8]. Still, knowledge of the brain complications in sepsis is limited, especially in deeply sedated patients, and brain monitoring is frequently neglected [9,10].

In this scenario, the introduction of brain ultrasonography (US), either blind (transcranial Doppler, TCD) or with 2D images integration (transcranial color-coded duplex sonography, TCCS), offers as a proxy for neuromonitoring in intensive care unit (ICU) with several advantages such as wide availability, possibility to assess multiple organs with the same instrument, and rapid responses at the bedside (point of care ultrasound, POCUS) [11,12]. Brain US can be used to monitor cerebral blood flow (CBF), evaluate non-invasive intracranial pressure (nICP), estimate midline shift, assess ventricles’ enlargement, and the eventual presence of masses or blood within the brain parenchyma. Thus, the use of brain US can give a panoramic overview of the brain parenchyma and its vessels [13,14].

However, evidence about the use of brain US in sepsis is limited. The aim of this scoping review is to summarize the current state of the literature focusing on three main questions: (1) is brain perfusion altered in sepsis? And if so, how is it altered? (2) Can we use brain US to guide sepsis resuscitation? (3) Can brain US predict the neurologic outcome of septic patients?

## 2. Materials and Methods

This scoping review follows the Preferred Reporting Items for Systematic Reviews and Meta-analysis protocol for scoping reviews (PRISMA-ScR) methods [15]. Furthermore, the proposed scoping review will be conducted in accordance with the JBI methodology for scoping reviews [16].

### 2.1. Search Strategy

The literature search was performed on 9 June 2024 in MEDLINE via PubMed, Scopus and Web of Science. A combination of “sepsis” OR “septic encephalopathy” OR “sepsis-associated encephalopathy” AND “brain ultrasound” OR “transcranial doppler” OR “transcranial color-coded doppler” OR “TCCS” was used. The same search strategy, including all identified keywords and indexed terms, has been adapted for each included database and/or information source. Other papers were retrieved from cited or related articles to these results. The full search syntax used for each database can be found in the Supplementary Materials (Attachment S1).

### 2.2. Eligibility Criteria

#### 2.2.1. Types of Participants

Studies on adults (>18 yo) were included. Articles on pediatric population, animal studies, and experimental sepsis studies were excluded. Studies regarding populations with intracranial infections were excluded.

#### 2.2.2. Concept

This review aims to assess the usefulness of brain US in critically ill septic patients, to determine if CBF alterations exist and can be used to predict the neurologic outcome or guide resuscitation. All the brain US metrics useful to answer the review questions will be taken into account and described.

#### 2.2.3. Context

Critically ill patients admitted to ICU for sepsis or septic shock regardless of sex, geographic location, or cultural differences.

#### 2.2.4. Type of Sources

All the articles in English published until 9 June 2024 were considered eligible for the review. Articles with full text not in English were excluded. Based on the study design, randomized controlled trials, clinical trials, and observational studies were included. Literature reviews were included only if they were systematic reviews with or without meta-analysis. Expert opinions, letters to the editor, guidelines, consensus, and editorials were excluded.

### 2.3. Source of Evidence Screening and Selection

**Figure 1:**
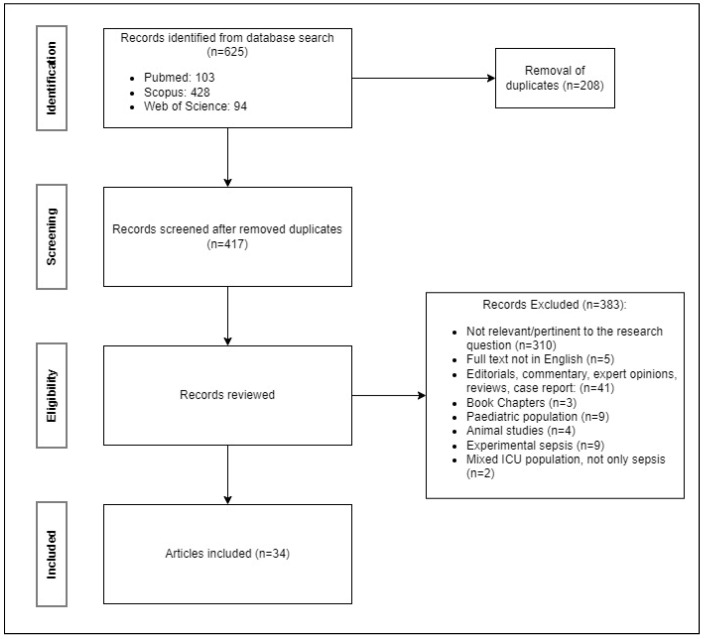
A quorum flowchart for articles selection.

All the articles were imported after searching in an Excel^®^ spreadsheet (Microsoft Corporation, Redmond, Washington, DC, USA). Duplicates were removed, and articles were progressively included or excluded blindly by two authors (GC and IC) in accordance with the previously cited criteria. Screening was based at first on titles and abstracts reading, and then on full text of the papers identified as potentially relevant. Any controversies between authors were resolved by discussion and opinion of a third author (FC). The inclusion process is shown in Figure 1.

### 2.4. Data Extraction Process

Data were extracted from the included papers by two independent reviewers, using a data extraction form independently developed by the authors. For each article we extracted aims, characteristics of the studied population (e.g., patients, age, setting), sample size, study design, methods, brain US metrics analyzed, time of observation, and the key findings and conclusions in terms of the previously mentioned outcomes.

Descriptive tables were used to summarize all the retrieved information from each study (Supplementary Materials, Attachment S2). The articles were tagged for subtopics based on the review questions and summary tables were created for each subtopic. The authors discussed the results and updated the tables where needed in a continuous process.

## 3. Results

A total of 625 articles were scrutinized, of which 208 were duplicated. After application of the exclusion criteria, 34 articles were included for data extraction (Figure 1). In total, 85% of the articles were observational studies (of which 60% were cohort studies and 40% case-control studies) and 15% were systematic reviews with or without meta-analysis. No RCTs were found.

### 3.1. Perfusion Abnormalities in Sepsis

#### 3.1.1. Cerebral Blood Flow, Pulsatility Index, Resistance Index, Cerebrovascular Resistances, and Other Intracranial Haemodynamics Indexes Alterations

The feasibility of TCD insonation during sepsis was demonstrated by Pierrakos et al. [17] in a cohort of 20 patients where TCD was used for the estimation of CBF velocities, cerebrovascular resistances (CVR), and pulsatility index (PI) with a feasibility of 91% through the acoustic bone window.

Many studies evaluated CBF velocity in septic patients, highlighting various changes. Baseline middle cerebral artery velocities (MCAv) were reported either lower [18], non-different [19], or higher [20] in respect to healthy controls or other anesthetized patients. Regarding CVR, these might be affected by multiple factors, but they were reported as reduced [21], but still functionally [22], even if the vasoconstrictor response might be reduced and slower with a difficulty in reversing after the vasoconstrictor stimulus has ceased [18,19].

Straver et al. [23] observed an inverse relationship between the systemic vascular resistance index and MCAv, with abnormalities in middle cerebral artery (MCA) and internal carotid artery (ICA) flow velocities being more pronounced in severe sepsis. These abnormalities were especially notable in non-survivors, who exhibited higher CBF and ICA velocities, in addition to an MCA/ICA index often >2, suggesting that a mild vasospasm can occur in basal cerebral arteries.

Pierrakos et al. [17] further supported the observation that cerebral vascular constriction occurs early in sepsis and is detectable by TCD. This constriction, reflected in elevated PI and Resistance Index (RI), suggests that CBF alterations are a common feature in the early stages of sepsis. PI and RI were analyzed in multiple studies, reporting that its value is often higher in patients with sepsis [Table 1]. In comparison to healthy controls, Szatmàri et al. [18] reported higher PI in accordance with the two following studies [17,19] even if the PI values were within the normality range (<1.3). In accordance with these findings, additional studies reported higher but <1.3 PI in sepsis than in controls [24,25] with the most frequent alterations in the first stages of sepsis [26,27]. However, it is important to notice that PI is not only a CVR estimator but is influenced by multiple vessel properties such as compliance, perfusion pressure, and heart rate [28].

A systematic review and meta-analysis from 2017 [29] evaluated TCD studies in septic patients to identify the cerebral hemodynamic course of the disease and analyze the cerebral hemodynamic parameters. They found that in early sepsis, median MCAv and PI were increased, while cerebral autoregulation (CAR) remained unchanged. In later sepsis, median MCAv normalized, PI reduced, and CAR became impaired. In addition, they stated that increased PI may indicate higher CVR in sepsis.

A particular note must be given to the cerebral circulation time (CCT) that uses contrast-enhanced ultrasound to calculate the transition time from the internal carotid artery to the internal jugular vein. This time has been identified as an independent predictor for SAE with an AUC of 0846. Its calculation, however, requires a specific software and a trained US operator.

**Table 1:** A summary of findings for studies regarding cerebral perfusion alterations. CBFi: cerebral blood flow index. CCP: critical closing pressure. CCT: cerebral circulation time. CD: cognitive decline. CO_2_R: CO_2_ reactivity. CRC: cerebrovascular reserve capacity. CVR: cerebrovascular resistances. ICA: internal carotid artery. MAP: mean arterial pressure. MCAv: middle cerebral artery velocities. PI: pulsatility index. RI: resistance index. SAD: sepsis-associated delirium. SAE: sepsis-associated encephalopathy. TCD: transcranial doppler.

Study	Main Findings	Metric Used	Sample Size (Septic Patients)
Straver 1996 [23]	Inverse relationship between systemic vascular resistance index and mean and diastolic MCAv. MCA/ICA index and MAP showed an inverse relationship (changes in MCAv were more pronounced than changes in ICA). MCA and ICA flow velocities abnormalities are more pronounced in severe disease and in non-survivors.	MCAv; PI; MCA/ICA index	20
Thees 2007 [30]	CO_2_R seemed not to be impaired. They did not observe abnormal findings explaining neurological abnormalities. CCP increased as expected during hyperventilation (25 ± 11 to 39 ± 15 mmHg).	CO_2_R, CCP; CBF calculated with thermodiluition and indocyanine green dye; CMRO_2_	10
Pfister 2008 [31]	In total, 12/16 patients presented SAD. There were no differences in CBF between SAD and non-SAD groups.	MCAv, Mx	16
Szatmàri 2010 [18]	PI was higher in the group with sepsis. Vasomotor response was slower and lower in sepsis (less CRC and lower systolic MCAv).	PI, acetazolamide test, cerebrovascular reactivity, CRC	14
Fülesdi 2012 [19]	PI was higher in septic patients. CRC was similar in the two groups while cerebrovascular reactivity decreased slower in the septic group (more prolonged vasodilatory response).	Acetazolamide test, cerebrovascular reactivity, CRC	16
Pierrakos 2013 [17]	TCD has a feasibility of 91% vs. 85%; *p* = 0.89 (septic vs. controls) due to acoustic bone window. PI and RI were higher in patients with sepsis than controls and higher in the first day. Cerebral vascular constriction is detectable by TCD in the early stage of sepsis.	MCAv, PI, RI, eCBF	20
Pierrakos 2014 [26]	PI on the first day was a good predictor of the presence of confusion (AUC = 0.908, 95%, CI 0.80–0.98, *p* < 0.01). For a cut-off value of 1.3, there was a 95% sensitivity and an 88% specificity.	PI	40
Toksvang 2014 [32]	The increase in MAP with noradrenaline generated a mean increase in MCAv of 14% (2–22%). There was poor agreement between TCD and NIRS for CBF estimation.	MCAv	8
Berg and Plovsing 2016 [22]	Hyperventilation was associated with a 36% increase in CVR, and a consequent 22% reduction in MCAv. CO_2_R is preserved in septic patients.	CVR, CO_2_R	16 (only 7 underwent hyperventilation)
Pierrakos 2017 [27]	PI was higher in patients with CD (2.2 ± 0.7 vs. 1.4 ± 0.5, *p* = 0.02) and CBFi was lower (363 ± 170 vs. 499 ± 133, *p* = 0.03). In univariate analysis, delirium and PI on the first day of the study were related to CD but in the multivariate analysis PI was not found to be related to CD independently of the presence of delirium.	PI, CBFi	28
Le Dorze 2018 [20]	Baseline CO and HR were higher, and MAP was lower in the sepsis group when compared to a brain injury and an anesthetized group of patients (controls). PSV was higher in the sepsis group than in the control group but not with the BI group. After a fluid challenge, PSV and EDV increased significantly only in the sepsis group. No significant correlations between systemic and cerebral hemodynamic changes were observed in any group.	PSV, EDV	38
Feng 2021 [24]	The SAD group exhibited lower levels of EDV and a higher PI but all within a normal range (0.98 ± 0.19 vs. 0.84 ± 0.20, *p* = 0.019).	MCAv, CBFi, PI, THRR	51
Zheng 2023 [21]	Patients with SAE showed significantly elevated PSV (107 [69–138] cm/s vs. 85 [69–101] cm/s, *p* = 0.002) and mean MCAv (57 [37–93] vs. 54 [42–66], *p* = 0.045) even if only in the left MCA and with mean MCAv within the normal range. The PI and RI were significantly higher in the SAE group than in the non-SAE group (even if the values were within the normal range). Patients with agitation had higher MCAv and lower PI and RI than patients with decreased consciousness, suggesting lower CVR.	MCAv, PSV, EDV, PI, RI, FV, CBF volume	198
Mei 2024 [25]	The SAE group displayed significantly elevated levels of PI, RI, and CCT, while EDV was lower. CCT emerged as the most efficacious predictor for SAE, with an AUC of 0.846. S100β, PI, and CCT were identified as the independent predictors for SAE.	PI, RI, CCT	67

#### 3.1.2. Autoregulation Estimation and Other Forms of Vessels’ Reactivity

When a single method to estimate CAR was used, septic patients variably presented altered or impaired autoregulation. Some studies reported near a half probability to find an altered CAR [33,34,35] while others reported normal CAR [36] [Table 2].

Some studies pointed out the role of the timing from sepsis onset to justify different states of autoregulation. A systematic review and meta-analysis conducted in 2017 [29] concluded that CAR remains unchanged in early sepsis, while it became impaired later. However, this study has drawn its conclusion from four studies evaluating CAR in the first 24 [36], 48 [31], and 72 h [37], and in an undetermined time from admission [38]. Unfortunately, the exact time of the measurements within those time spans were not reported. Conversely, a study by Schramm et al. [39] measured CAR throughout the first 4 days from admission and reported a decreasing incidence of impairment with a percentage going from 60% at day 1 to 46% at day 4.

Concerning the causative agents of CAR impairment, a study by Pfister et al. [31] found a significant association between delirium, elevated C-reactive protein, and impaired CAR, suggesting that inflammation could impede cerebrovascular endothelial function. Endothelial function was addressed as the translation causative mechanism, as inflammation “per se” was not associated with CAR impairment when measured by interleukin-6. On the other hand, two studies pinpointed the relevance of the mean arterial blood pressure (ABP) or arterial partial pressure of CO_2_ during CAR estimation stating that a weaker autoregulation might be detected when a low ABP or a high CO_2_ are present, since it has reached the lower limit of autoregulation [37,38]. As hypotension is a common clinical feature in sepsis, reduction of cerebral perfusion pressure (CPP) with consequent overtaking of the lower limit of autoregulation might be a frequent event that increases the chances to observe an impaired CAR.

Regarding CO_2_ reactivity (CO_2_R), some studies reported an impaired value [40,41], while others reported a normal value [30,36] [Table 3]. Interestingly, one study reporting an impaired CO_2_R did not find a relationship with mortality, while another study where CO_2_R was not impaired reported a pathological neurologic exam in all the survived patients enrolled [30].

**Table 2:** Studies regarding autoregulation in septic patients. CAI: cerebral autoregulation index. CAR: cerebral autoregulation. IOR: index of autoregulation. Mx, Mxa: mean flow index. THRR, THRT: transient hyperemia response ratio or transient hyperemia response test. SAE: sepsis-associated brain dysfunction. SAD: sepsis-associated delirium.

Study	Main Findings	Metric Used	Sample Size (Septic Patients)
Matta and Stow 1996 [36]	Mean IOR was 0.92 (intact autoregulation).	IOR	10
Pfister 2008 [31]	CAR was altered in the SAD patients, with no differences on perfusion in respect to the non-SAD group.	Mx	16
Steiner 2009 [38]	Correlation between Mx and another index of autoregulation from near infrared spectroscopy showed a strong positive association (R = 0.81; *p* < 0.0001). PaCO_2_-induced dilatation of flow-regulating vessels was associated with worse autoregulation.	Mx	23
Taccone 2010 [37]	CAR was impaired in 66% of patients, and impairment increased for higher PaCO_2_ values.	CAI	21
Schramm 2012 [39]	CAR was impaired in 88% of the patients, with a decreasing prevalence during the days (day 1—60%, day 2—59%, day 3—41%, day 4—46%). The status of CAR at day 1 was related to SAD development at day 4. SAD was associated with age.	Mx	30
Crippa 2018 [33]	A total of 50% of patients presented impaired CAR. There was no difference in Mxa between survivors and non-survivors (at ICU discharge). Mxa was higher in patients with SAE. The best Mxa cut-off to predict SAE was 0.18 (sensitivity 79%, specificity 47%).	Mxa	100
Feng 2021 [24]	The SAD group had a significantly higher level of cerebrovascular dysfunction (THRR index < 1.09, 40 vs. 10%, *p* = 0.01). A THRR index < 1.09 was a SAD predictor (OR = 5.77, 95% CI: 1.222–27.255, *p* = 0.027).	THRR	51
Crippa 2022 [34]	A total of 53% patients had impaired CA.	THRT	40
Caldas 2022 [35]	Median ARI and Mxa values were 4.38 [2.83–6.04] and 0.32 [0.14–0.59], respectively. Impaired CAR according to the ARI threshold was observed in 42% of patients; impaired CAR according to the Mxa threshold was observed in 53% patients. Mx and ARI had a weak correlation and a poor agreement to classify CAR.	ARI, Mx	95

In summary, variable rates of impaired CAR are reported in the literature. Even if some studies report normal CAR, the majority of studies report frequent CAR alterations and highlight the association of CAR impairment with SAD or other forms of impaired neurologic outcome in sepsis. Destruction of CAR is probably a phenomenon that comes and goes with different incidence during the course of the illness, with a high probability of alterations during the earlier and more severe phases. In addition, typical features of sepsis as hypotension or a high arterial CO_2_ might influence observations, increasing the rate of impaired CAR measures.

### 3.2. Resuscitation

A study by De Goede et al. [42] compared the MCA flow waveform between septic non-resuscitated patients and controls. Non-resuscitated patients presented lower diastolic and peak systolic MCAv, with a decreased acceleration time from baseline to the systolic peak. In addition, the absence of a secondary systolic peak, that progressively reappeared during resuscitation, was noticed. The acceleration time as well as the first and second systolic peak velocities increased significantly after resuscitation. The authors stated that brief repetitive TCD measurements during resuscitation were feasible and the reappearance of the second systolic peak could be used as a hemodynamic monitoring metric.

Another study [20] evaluated CBF modifications induced by fluid challenges in fluid responders. A comparison was made between septic patients, anesthetized patients, and brain injured patients. They found an increase in peak systolic velocity (PSV) and end diastolic velocity (EDV) of MCA only in the sepsis group with no significant correlations between systemic and cerebral hemodynamic changes in any group, drawing to the conclusion that the increase in cardiac output after a fluid challenge elicits an increase in MCAv only in patients with severe acute systemic inflammation. This might be due to the sepsis-induced impairment of CAR or because the lower limit of autoregulation is often undertaken in this population. However, since CBF changes were not linked to systemic hemodynamics, direct CBF monitoring during resuscitation may be crucial to provide an adequate brain perfusion.

Regarding the possibility of an increase in cerebral edema due to aggressive fluid resuscitation, Pfister et al. [43] observed no correlation between the nICP estimation with TCD and the daily fluid administration or balance.

However, due to the paucity of studies regarding resuscitation, the heterogeneity of the populations and timing from admission makes it impossible to recommend the routine use of brain US during sepsis resuscitation.

### 3.3. Non-Invasive Cerebral Perfusion Pressure and Estimation of Intracranial Pressure

Unfortunately, ICP cannot be directly measured in these patients as they are considered too harmful. The reliability of ICP estimation with TCD, however, has been demonstrated to be low on the absolute value, but very good to reflect the general trend in comparison with the invasive ICP [44]. However, considering the incidence of hypotension and hypoperfusion occurring during sepsis and septic shock, the estimation of non-invasive cerebral perfusion pressure (eCPP) by the means of brain US can be a useful monitoring tool.

Only three studies regarding nICP and estimated cerebral perfusion pressure (eCPP) assessment in sepsis were found (Table 4). The feasibility of monitoring changes in cerebral perfusion with TCD during sepsis was assessed by Pierrakos et al. in 2013 [17]. A study by Pfister et al. [43] evaluated eCPP and therefore nICP in 16 septic patients, with daily repeated measures, hypothesizing that aggressive fluid administration with a derangement of vascular permeability could have provoked cerebral edema and increased ICP. However, the nICP was always below 20 mmHg, and the absolute value of nICP was not related to mortality. Nevertheless, patients with low eCPP presented higher levels of S-100β protein, suggesting that brain damage was directly related to hypoperfusion rather than intracranial hypertension. Another study [34] estimated eCPP and nICP in 40 septic patients. In total, 55% of patients presented an eCPP below 60 mmHg with a nICP greater than 16 mmHg. Some patients in the study presented a nICP around 40 mmHg, and a higher value of nICP was related to a lower neurological pupil index. The same group of researchers analyzed eCPP and nICP in a cohort of 132 patients [45], highlighting again that even if the majority of septic patients (63%) presented with normal eCPP and nICP, nearly one third had low eCPP values. No differences were found on the incidence of SAE between patients with abnormal eCPP or nICP and those who had normal values.

### 3.4. Evaluation of the Neurologic Outcome

SAE has classically been associated with delirium during its acute phases. The majority of studies evaluated delirium in ICU using the CAM-ICU scale; concerning long-term neurologic outcomes, there is a paucity of studies, and we identified only one cognitive decline (CD) related study [27] (Table 5).

A study by Feng et al. [24] evidenced how an altered transient hyperemia response test (THRT) evaluated with TCCS in the first 6 h after resuscitation in patients with shock, was an independent predictor of SAD [OR 5.77]; patients with delirium presented a poorer outcome (survival at 28 days), a higher APACHE II score, increased biomarkers for neuronal damage (NSE, neurospecific enolase), increased ICU length of stay, and more days of ventilation. In a similar study [39], daily evaluation of CAR during the first 4 days after sepsis was assessed; patients that presented impaired autoregulation (Mx > 0.3) at day 1, had a higher incidence of delirium at day 4. However, in this case, association with severity of the illness (APACHE II) or neuronal damage markers (NSE) was not evidenced. Another study, in accordance with the precedents, demonstrated that CAR derangement was associated with delirium, independently of APACHE II score, MAP, CBF velocity, and catecholamine requirements [31].

Interestingly, in a study by Pierrakos et al. [27], an ROC curve analysis showed that PI on the first day (but not on the third day) was a good predictor of the presence of confusion (AUC = 0.908, 95%, CI 0.80–0.98, *p* < 0.01). PI was related to confusion independently from age or APACHE II score. In another study from the same group [27], 50% of patients that presented CD or persistent coma at ICU discharge presented an elevated PI and a lower CBFi on the first day of admission for sepsis (PI 2.2 ± 0.7 vs. 1.4 ± 0.5, *p* = 0.02); PI increase was persistent in patients with persistent coma, while the alterations of PI were momentaneous in the other patients. Crippa et al. [33] identified as SAE independent predictors an impaired CAR (evaluated with Mxa), vascular diseases, and mechanical ventilation. The best Mxa cut-off to predict SAE was 0.18 (sensitivity 79%, specificity 47%). However, Mxa was not different between survivors and non-survivors.

Regarding possible increases in nICP, two studies failed in proving its role for SAE and mortality prediction [45,46]. Even if Czempik et al. [46] found a high probability of measuring a ONSD > 5.7 at least once during the ICU stay, these high measurements were not related to CRP concentrations, highest daily lactate, or SOFA; assuming that a dilated ONSD could be related to SAE, the authors concluded that ONSD measurement must be used cautiously for SAE screening. In the other study by Crippa et al. [45], patients with normal and high nICP or normal and low eCPP presented no difference in SAE occurrence or mortality.

A recent study [25] tried to use a composite TCD and biohumoral input to predict SAE. After analyzing various metrics as inputs, they concluded that S100β, PI, and CCT measured in the first 24 h from ICU admission were independent predictors for SAE.

Finally, three systematic reviews and meta-analyses that investigated if delirium was related to microvascular damage in sepsis concluded that cerebral perfusion disturbances might be associated with SAD. However, heterogeneity in delirium definitions and assessment in addition to different TCD metrics analyzed can be confounding factors [47]. In the same way, another systematic review concluded that the reviewed studies indicate a link between impaired CAR and poor outcomes, but due to variations in study design and methodological limitations, further research is needed [48]. Heterogeneity in neuromonitoring tools in sepsis was also found in another study [49] and thus, conclusions about the impairment of the outcome and predictive indexes are inconsistent, even because alterations in the metrics found were often negligible (i.e., within the normal values).

## 4. Discussion

The main findings of the present manuscript are as follows: (1) CBF alterations are common in septic patients and (2) might be related to sepsis severity. (3) TCD metrics can be used to predict neurologic impairment and (4) brain perfusion monitoring during resuscitation is feasible. However, the body of evidence we found was constituted only by observational studies (85%) or systematic reviews with/without meta-analysis (15%), often with small sample sizes and with very heterogeneous methodologies. For this reason, the reproducibility of results might be impaired.

Regarding studies heterogeneity we found some main criticalities regarding sepsis definition (i.e., study populations), time of observation, and metrics considered. An overview of the sepsis definitions used, and distribution of studies is available in the Supplementary Material, Attachment S3. The different sepsis definition has influenced the composition of the study cohorts, modifying the inclusion criteria. Nevertheless, even if sepsis definition might have had a minor impact on patients’ selection, the presence of shock might have had more influence on the observed cerebral perfusion abnormalities, as highlighted in one of the included studies [38]. In fact, the cohorts of the studies were often composed of a mix of septic and septic shock patients.

The time of observation varied among studies, with studies observing patients within 24, 48, or 72 h, or in a not specified timeframe; even the relationship between observation and time 0 was different, with some studies referring to symptoms’ onset and others to ICU admission. The observation time represents a crucial methodological issue because CBF alterations might present at different timepoints during the illness as presented in the study by Schramm et al. [39]. The possibility to have multiple observations during the days or even multiple observations in the same day, could thus be a big improvement for results interpretation.

Concerning the metrics analyzed, a discussion of multiple ways of calculating similar indexes is beyond the aim of the manuscript and has been addressed elsewhere [50]. An overview of all the brain US metrics used by authors of the 34 included papers is available in Table 6. The variation of the metrics comes from the lack of standardization of the perfusion indexes, except for the velocity’s evaluation and resistance indexes (e.g., RI and PI). In this perspective, the publication of a white paper and alignment on this topic might be of great benefit for further research studies aiming at evaluating brain perfusion abnormalities in sepsis. In fact, readers should bear in mind that there is a huge difference in the way of calculating indexes of autoregulation or non-invasive ICP or CPP. In respect to CAR metrics, we can divide these indexes into qualitative and quantitative, that are typically either snapshot or continuously calculated metrics (Table 7). The concordance of different indexes is anything but obvious, with some studies demonstrating poor agreement between different indexes [35]. It remains unclear whether the poor correlation between CAR metrics is due to inherent differences in the various algorithmic models or if it results from unidentified external “noise” present in the real-world data [51], and this uncertainty complicates meaningful comparisons.

Other sources of heterogeneity must be cited for what concerns nICP estimation. In this case, not only different methods of estimating it were used, but one of the most used methods of calculation was able to reflect with high reliability the trend of the real ICP; however, it has a high confidence interval in estimating the absolute value [44].

Some limitations of the present manuscript are intrinsically related to the operator dependency of the echographic technique, and to the nature of scoping reviews, which try to answer broad spectrum questions with highly heterogeneous studies. In addition, the low quality of evidence represents another limitation.

### Research Gaps, Awaited Studies, and Future Directions

More efforts need to be made in establishing with certainty cerebral haemodynamic alterations in sepsis, and how they may change during the critical illness course. In addition, there is a lack of studies on resuscitation and non-invasive ICP and CPP estimation that could be useful in a systematic assessment of brain health and might contribute to ruling out intracranial pathologies whenever neurologic impairment is present. Implementation of 2D brain images retrievable with TCCS (ONSD, midline shift, direct visualization of third and lateral ventricles) might be a valuable addition to the nICP and eCPP estimation when a proper acoustic bone window is available [74]. Even if these techniques have been extensively described, at the moment, no studies have systematically evaluated their usefulness and impact on the management of septic patients in ICU [75].

Concerning resuscitation, only general protocol for patients with shock [11,76] or POCUS protocols not involving the brain have been published [77], and research regarding peripheral perfusion-guided resuscitation is awaited [78,79]. In this perspective, studies assessing brain perfusion in traumatic brain injured patients demonstrated that targeting a perfusion pressure within the range of autoregulation is feasible, and can be done at the bedside with proper instrumentations and the aid of a bedside software (ICM+ v 9.2.4.6, Cambridge enterprise, Ltd., Cambridge, UK) [80] even if the benefit of MAP titration to fit into the range of autoregulation has not yet been clearly established. For certain, the tailoring of therapies and the titration of drugs based not only on central haemodynamics but also on peripheral perfusion is promising and could be one of the critical interventions that dramatically improves the outcome of septic patients as one MAP target is not always adequate for all [79,81,82]. The implementation of brain multimodal evaluation including brain US and other non-invasive neuromonitoring tools (i.e., near infrared spectroscopy, pupillometry, and EEG), may give additional information about brain hypoperfusion, electric malfunctioning (i.e., epilepsy), sedation adequacy, and prediction of outcome, and might be useful in clinical practice [83,84].

Studies evaluating the role of brain US should be more standardized and should observe the patients during the days to assess if more phenotypes of CBF alterations exists and how the course of illness can modify these alterations. Prospectively, new findings could suggest new clinical strategies to protect the brain during sepsis and that might guide a controlled trial aiming at maintaining brain perfusion during resuscitation.

## 5. Conclusions

The current body of evidence regarding brain US in patients with sepsis and septic shock has a poor grade and the small sample sizes may affect the generalizability of the conclusions; thus, the current evidence is not sufficient to support the routine use of brain US in sepsis. However, brain US is a promising tool able to identify cerebral perfusion alterations and is feasible at the bedside. Use of brain US during resuscitation might help in ensuring an adequate brain perfusion and improve the outcome. Standardization of the metrics for further studies is critical to establish the usefulness of brain US and its capacity to modify the clinical management.

## Figures and Tables

**Table 3:** A summary of findings for studies regarding CO_2_ reactivity and the acetazolamide test. CO_2_R: CO_2_ reactivity. NCR: normalized CO_2_ reactivity. CAR: cerebral autoregulation. CRC cerebrovascular reserve capacity.

Study	Main Findings	Metric Used	Sample Size (Septic Patients)
Matta and Stow 1996 [36]	CO_2_R was within normal limits for all patients.	CO_2_R	10
Terborg 2001 [40]	During septic shock, NCR was significantly reduced.	NCR	8
Bowie 2003 [41]	CO_2_R was abnormal in 10/12 patients. This was not related to mortality or other clinical parameters.	CO_2_R	12
Thees 2007 [30]	CO_2_R was not impaired. However, the survivors showed a pathologic neurological examination.	CO_2_R	10
Berg and Plovsing 2016 [22]	CO_2_R is preserved in sepsis. Short-term hyperventilation does not necessarily enhance CAR.	Phase, gain, coherence	16 (only 7 underwent hyperventilation)
Szatmàri 2010 [18]	Vasomotor response was slower and lower in sepsis (less CRC and lower systolic MCAv).	Acetazolamide tes, CVR, CRC	14
Fülesdi 2012 [19]	CRC was similar in the two groups while CVR decreased slower in the septic group (more prolonged vasodilatory response).	Acetazolamide tes, CVR, CRC	16

**Table 4:** A summary of results of the studies regarding non-invasive ICP (nICP) and estimated cerebral perfusion pressure (eCPP). THRT: transient hyperemia response test.

Study	Main Findings	Metric Used	Sample Size (Septic Patients)
Pfister 2008 [43]	A total of 47% of patients showed nICP > 15 mmHg in at least one day. nICP increases were moderate and never exceeded 20 mmHg. nICP was strongly correlated with MAP but did not differ between survivors and non-survivors. A total of 73% of patients had eCPP < 60 mmHg (20% falling <50 mmHg). Low eCPP was associated with high S-100β levels. There was no link between nICP and fluid administration.	nICP, eCPP	16
Crippa 2022 [34]	A total of 53% of patients had impaired CAR, 55% had low eCPP, and 38% had high nICP. Low eCPP and high nICP was seen in 35% of patients. Pupillary dilation velocity was significantly lower in those with impaired CAR. Patients with low eCPP or high nICP had lower Neurological Pupil index (NPi) values.	THRT, nICP, eCPP	40
Crippa 2023 [45]	The median eCPP was 63 mmHg, with 33% having low eCPP. The median nICP was 8 mmHg, with 4% having high nICP. Most patients (65%) had normal eCPP and nICP; 31% had low eCPP with normal nICP; 2% had low eCPP and high nICP; 2% had normal eCPP and high nICP. There were no significant differences in SAE occurrence or in-hospital mortality between patients with altered eCPP or nICP compared to those with normal values.	eCPP, nICP, Mxa.	132

**Table 5:** A summary of studies for neurologic outcome prediction. CAR: cerebral autoregulation. CBFi: cerebral blood flow index. CCT: cerebral circulation time. CD: cognitive decline. CRP: C-reactive protein. eCPP: estimated cerebral perfusion pressure. nICP: non-invasive intracranial pressure. MCAv: middle cerebral artery velocities. Mx: mean flow index. ONSD: optic nerve sheet diameter. PI: pulsatility index. SAD: sepsis-associated delirium.

Study	Main Findings	Metric Used	Sample Size (Septic Patients)
Pfister 2008 [43]	No significant correlations between nICP, daily change in nICP, or relative change in nICP and overall or daily fluid administration or balance.	MCAv, nICP, eCPP	16
Pfister 2008 [31]	Mx was altered in SAD patients. No differences in CBF between the SAD and non-SAD group.	MCAv, Mx	16
Schramm 2012 [39]	Twenty-five patients (88%) showed impaired CAR during the four days with a decreasing prevalence during days (day 1—60%, day 2—59%, day 3—41%, day 4—46%). Delirium developed in 76% of patients. The status of CAR at day 1 was related to the development of delirium at day 4.	Mx	30
Pierrakos 2014 [26]	Twenty-one patients (55%) presented delirium (positive CAM-ICU test). ROC curve analysis showing only PI on the first day and not the third day was a good predictor of the presence of confusion (AUC = 0.908, 95%, CI 0.80–0.98, *p* < 0.01). PI was related to confusion independently from age or APACHE II score.	MCAv, PI, CBFi	40
Pierrakos 2017 [27]	Fourteen patients (50%) presented CD at the time of discharge. Only on the first day of the study PI was higher in patients with CD (2.2 ± 0.7 vs. 1.4 ± 0.5, *p* = 0.02) and CBFi was lower (363 ± 170 vs. 499 ± 133, *p* = 0.03). In univariate analysis, delirium and PI on the first day were related to CD (OR: 36.1, 95%CI 4.3–299.1, *p* = 0.01, OR:4.1, 95%CI 1.1–15.2, *p* = 0.03), but in the multivariate analysis PI was not found to be related to CD independently of the presence of delirium.	MCAv. PI, CBFi	28
Crippa 2018 [33]	There was no difference in Mxa between survivors and non-survivors at ICU discharge. SAE was more common in patients with altered CAR than in those with intact CAR (34 of 50 [68%] vs. 23 of 50 [46%]; *p* = 0.04), and Mxa was higher in patients with SAE (0.47 [0.21–0.64] vs. 0.23 [−0.12–0.52]; *p* < 0.01). In multivariable analysis, higher Mxa, vascular disease, and mechanical ventilation were independent predictors of SAE. The best Mxa cut-off to predict SAE was 0.18 (sensitivity 79%, specificity 47%).	Mxa	100
Czempik 2020 [46]	A total of 49/80 ONSD measurements exceeded 5.7 mm. There were no correlations between ONSDs and CRP concentrations, highest daily lactate, or SOFA. ONSD measurement should be applied for screening of SAE cautiously.	ONSD	10
Feng 2021 [24]	The logistic regression analysis demonstrated that several independent risks were SAD predictors: rSO_2_ <55% [OR = 3.864, 95% CI: 1.026–14.550, *p* = 0.046] and the THRR index < 1.09 [OR = 5.77, 95% CI: 1.222–27.255, *p* = 0.027]. Patients with SAD have a close correlation with poor outcomes.	MCAv, CBFi, PI, THRR	51
Crippa 2023 [45]	SAE occurrence and mortality did not differ between patients with low and normal eCPP or between patients with high and normal nICP.	eCPP, nICP, Mxa	132
Mei 2024 [25]	The SAE group displayed significantly elevated levels of NSE, S100β, PI, RI, and CCT, while EDV was lower (all *p*-values < 0.05). CCT emerged as the most efficacious predictor for SAE, with an AUC of 0.846. S100β, PI, and CCT were identified as independent predictors for SAE.	MCAv, PSV, EDV, PI, RI, CCT	67

**Table 6:** An overview of the brain US metrics used by authors and their reported definitions. CAR: cerebrovascular autoregulation. CBF: cerebral blood flow. dCA: dynamic cerebral autoregulation. EDV: end diastolic velocity. FV: flow velocity. MCAv: middle cerebral artery flow velocity. MAP: mean arterial pressure. PSV: peak systolic velocity.

TCD/TCCS Metrics	Acronym	Index Explanation	Reference for Calculation	Article
Acceleration	acc	Acceleration is defined as the maximal increase in FV per second during the systolic upstroke and was obtained by taking the maximum of the first order derivative of the ensemble average during the period lasting from systolic onset until first local maximum.	Schaafsma A. Improved parameterization of the transcranial Doppler signal. Ultrasound Med Biol 2012;38:1451–1459. [52]	De Goede 2017 [42]
Autoregulation index	ARI	The signals were filtered, interpolated and resampled at 5 Hz. Then, the Welch method was used for smoothing spectral estimates derived from the fast Fourier transform (FFT) over segments of 102.4 s with 50% overlap. ARI values were obtained by fitting a second-order polynomial to minimize the error, using neighboring integer ARI values as a reference. ARI ranges from 0 (absent dynamic cerebral autoregulation, dCA) to 9 (most efficient dCA).	Caldas et al., Dynamic autoregulation is impaired in circulatory shock. Shock Augusta Ga. (2020) 54:183–9. [53] Czosnyka et al., Monitoring of cerebral autoregulation. Neurocrit Care. (2014) 21(Suppl. 2):S95–102. [54] Panerai RB. Transcranial Doppler for evaluation of cerebral autoregulation. Clin Auton Res Off J Clin Auton Res Soc. (2009) 19:197–211. [55]	Caldas 2022 [35]
Cerebral autoregulation index	CAI	The ratio of the relative changes in cerebrovascular resistances (CVR) and MAP $CAI=\frac{\Delta MAP\%}{\Delta CVR\%}$ Normal value: 0–2.	Bouma GJ, Muizelaar JP. Cerebral blood flow, cerebral blood volume, and cerebrovascular reactivity after severe head injury. J Neurotrauma. 1992 Mar;9 Suppl 1:S333-48. [56]	Taccone 2010 [37]
Cerebral capillary closing pressure	CCP	Zero-flow velocity pressure as extrapolated by regression analysis of arterial pressure/MCAV plots, averaged over two respiratory cycles.	Thees C, Scholz M, Schaller C, Gass A, Pavlidis C, Weyland A, Hoeft A: Relationship between intracranial pressure and criti cal closing pressure in patients with neurotrauma. Anesthesiology 2002, 96:595-599 [57]	Thees 2007 [30]
Cerebral circulation time (assessed via contrast -enhanced ultrasound)	CCT	Similarly to TCD, CCT measures the interval between the entry of arterial blood in the internal carotid artery (ICA) and its exit through the internal jugular vein (IJV). Utilizing a C5-1 convex array transducer, both the ICA and IJV were visualized in a transverse cross-sectional plane, specifically at a location 1.5 cm superior to the bifurcation of the common carotid artery. Settings were switched to “contrast mode” with reduced mechanical and thermal indices. An FDA-approved microbubble contrast agent (SonoVue, Bracco, Milan, Italy) was prepared in 5 mL of isotonic saline and rapidly administered via the median cubital vein, followed by a 5 mL saline flush. Bolus administration and subsequent CCT assessments were performed on the side demonstrating higher blood flow velocity in earlier TCD measurements. Analysis of the imaging data was executed through uninterrupted video capture, with time-intensity curves being isolated post-recording by a seasoned ultrasonographer. The inbuilt software automatically processed these curves after targeting the ICA and IJV.	Liu X, et al. A new method of measurement of cerebral circulation time: contrast-enhanced ultrasonography in healthy adults and patients with intracranial shunts. Ultrasound Med Biol. (2014) 40:2372–8. [58]	Mei 2024 [25]
Cerebral metabolic rate of oxygen	CMRO_2_	${CMRO}_{2}=CBF\cdot ({PaO}_{2}-{PvO}_{2})$ With PvO_2_ as the pressure of oxygen in the jugular vein.	-	Thees 2007 [30]
Cerebrovascular reserve capacity	CRC	The maximal % increase of the blood flow velocity after acetazolamide administration. $CRC=\frac{{MCA}_{v\;acz\;max}-{MCA}_{v\;rest}}{{MCA}_{v\;rest}}$	-	Szatmári 2010 [18], Fülesdi 2012 [19]
CO_2_ reactivity	CRCO_2_	The difference between the MCAv at hypocapnia and hypercapnia expressed as a percentage of the baseline MCAv per kPa change in ETCO_2_.	-	Bowie 2003 [41]
		Absolute CO_2_R: change in MCAv per kPa change in *P*aCO_2_. Relative CO_2_R: percentage change in MCAv at PaCO_2_ 5.3 kPa per kPa change in PaCO_2_.		Matta and Stow 1996 [36]
		Percentage change in MCAv per kPa change in PaCO_2_.		Thees 2007 [30]
CO_2_ reactivity, normalized	NCR	% change in CBF velocity per 1% increase in EtCO_2_.		Terborg 2001 [40]
Cerebrovascular resistances	CVR	CVR = MAP/MCAv	-	Taccone 2010 [37], Berg 2016 [22]
Cerebrovascular reactivity	CVR	CVR = (MCA_acz_ − MCAv _rest_)/MCAv _rest_; MCAv_acz_ is the MCA mean blood flow velocity measured at 5, 10, 15, and 20 min after acetazolamide, and MCAv_rest_ is the MCA mean blood flow velocity measured at rest.	-	Szatmári 2010, [18] Fülesdi 2012 [19]
Diastolic FV	Dias@560	Dias@560 was obtained by calculating the mean blood FV during the interval 520–600 ms after stroke onset. Finally, the acc, sys1 and sys2 values were divided by the dias@560 value for normalization.	Schaafsma A. Improved parameterization of the transcranial Doppler signal. Ultrasound Med Biol 2012;38:1451–1459. [52]	De Goede 2017 [42]
Estimated CBF (CBF index)	CBFi or CBF	$eCBF=MAP\cdot \frac{10}{1.47^{PI}}$	-	Pierrakos 2013 [17]
			Pierrakos 2013 [17]	Pierrakos 2014 [26]
			Pierrakos 2013 [17]	Pierrakos 2016 [27]
			-	Feng 2019 [24]
		A 25 mg dose of indocyanine green dye, dissolved in 40 mL of iced 5% glucose solution, was used as a double-indicator and injected into the right atrium through a central venous line. Dilution curves for both the dye and temperature were recorded simultaneously using thermistor-tipped fiber-optic catheters placed in the aorta (via a 30 cm catheter inserted into the femoral artery) and the jugular bulb. All measurements were taken from the sonographically controlled dominant (right) internal jugular vein. CBF was calculated based on the mean transit time of the first pass of the thermal and dye indicators using a specialized computer system.	Wietasch GJK, et al. Bedside assessment of cerebral blood flow by double-indicator dilution technique. *Anesthesiology* 2000, 92:367-375.13. [59] Mielck F, et al. Reliability of cerebral blood flow measurements by transcerebral double-indicator dilution technique. *Eur J* *Anaesth* 2001, 18:653-661. [60]	Thees 2007 [30]
Estimated CPP	eCPP	$eCPP=MAP\cdot \frac{EDV}{FVm}+14$	Czosnyka et al., Cerebral perfusion pressure in head-injured patients: A noninvasive assessment using transcranial Doppler ultrasonography. J. Neurosurg. 1998, 88, 802–808. [44]	Crippa 2022 [34], Crippa 2024 [35]
			Schmidt et al., Adaptive noninvasive assessment of intracranial pressure and cerebral autoregulation. Stroke. 2003 Jan;34(1):84-9. [61]	Pfister 2008 [43]
Intravascular flow volume	FV	For a defined vessel, FV was defined as the product of time-averaged flow velocity (TAV) and its cross-sectional area (A) according to the formula: $FV=TAV\cdot A=TAV\cdot [{\left(\frac{D}{2}\right)}^{2}\cdot \pi ]$ D= diameter. The CBF volume was determined as the sum of the FVs of the internal carotid artery and vertebral artery of both sides.	Scheel et al., Color duplex measurement of cerebral blood flow volume in healthy adults. Stroke 2000; 31:147–150 [62]	Zheng 2024 [21]
Index of autoregulation	IOR	The ratio of percentage change in estimated cerebral vascular resistance (CVRe) to percentage change in MAP, using the equations CVRe = MAP/MCAv and IOR =%∂CVRe/%∂ MAP, where MAP is at the time of MCAv measurement.	Matta BF, Lam AM, Strebel S, Mayberg TS. Cerebral pressure autoregulation and CO_2_-reactivity during propofolinduced EEG suppression. British Journal of Anaesthesia 1995; 74: 159–163. [63]	Matta and Stow 1996 [36]
Mean flow index	Mx or Mxa	General definition: the Mx or Mxa index is calculated as a moving correlation coefficient between short-term fluctuations in two signals over a specific time window (e.g., 5–10 s). Mx usually refers to a calculated index between CPP and MCAv; conversely Mxa refers to ABP and MCAv. In septic patients, thus, Mxa is used, even if in the papers it is commonly referred to as Mx or Mxa, alternatively. A positive correlation suggests that increases in blood pressure leads to increases in MCAv, indicating impaired autoregulation (Mxa > 0.3). In contrast, a near-zero or negative correlation indicates effective autoregulation, where CBF remains stable despite changes in MAP.		
		In this article: values of MAP and FV averaged every 10”. Mx is calculated every 60” as the moving linear correlation coefficient between the last 30 consecutive values of MAP and FV (5 min).	Piechnik SK, et al. The continuous assessment of cerebrovascular reactivity: a validation of the method in healthy volunteers. Anesth Analg 1999, 89:944-949. [64]	Pfister 2008 [31]
		In this article: values of MAP and FV averaged every 6”. Mx is calculated every 60” as the moving linear correlation coefficient between the last 30 consecutive values of MAP and FV (3 min).	Czosnyka et al., Monitoring of cerebral autoregulation in head-injured patients. Stroke. 1996;27:1829–34. [65] Piechnik SK et al., The continuous assessment of cerebrovascular reactivity: a validation of the method in healthy volunteers. Anesth Analg. 1999;89:944–9. [64]	Schramm 2012 [39]
		In this article: values of MAP and FV averaged every 10”. Mx is calculated every 60” as the moving linear correlation coefficient between the last 30 consecutive values of MAP and FV (5 min).	Czosnyka et al., Monitoring of cerebral autoregulation in head-injured patients. Stroke. 1996;27:1829–34. [65] Piechnik SK et al., The continuous assessment of cerebrovascular reactivity: a validation of the method in healthy volunteers. Anesth Analg. 1999;89:944–9. [64]	Steiner 2009 [38], Caldas 2022 [35], Crippa 2022 [2], Crippa 2024 [45]
		The Pearson’s correlation coefficient between the averaged ABP and flow velocity averaged on 10 s-consecutive windows with 50% overlap.	Czosnyka et al., Monitoring of cerebral autoregulation in head-injured patients. Stroke. 1996;27(10):1829–34. [65]	Crippa 2018 [33]
Non-invasive ICP or estimated ICP	nICP or eICP (Crippa 2022, Crippa 2024)	The mathematical algorithm built up starting from various TCD waveform parameters and ABP, that aims to estimate with precision the nICP.	Schmidt et al., Adaptive noninvasive assessment of intracranial pressure and cerebral autoregulation. Stroke. 2003 Jan;34(1):84-9. [61]	Pfister 2008 [43]
		$eICP=MAP\cdot \left(1-\frac{EDV}{FVm}\right)-14$	Czosnyka, M. et al. Cerebral perfusion pressure in head-injured patients: A noninvasive assessment using transcranial Doppler ultrasonography. J. Neurosurg. 1998, 88, 802–808. [44] Rasulo FA, et al. The accuracy of transcranial Doppler in excluding intracranial hypertension following acute brain injury: a multicenter prospective pilot study. Crit Care. 2017;21(1):44. [66]	Crippa 2022 [2], Crippa 2024 [45]
Resistance index	RI	RI = (PSV − EDV)/PSV		Berg 2015 [67], Berg and Plovsing 2016 [22], Caldas 2022 [35], Zheng 2023 [21], Mei 2024 [25]
Systolic component 1 and 2	Sys1 and Sys2	Sys1 and sys2 are the maximal flow velocities within the first and second systolic peaks and were obtained by taking the zero-line crossing of the first (if necessary second) order derivative of the ensemble average during the first 100 ms and during the remaining part of systole, respectively.	Schaafsma A. Improved parameterization of the transcranial Doppler signal. Ultrasound Med Biol 2012;38:1451–1459. [52]	De Goede 2017 [42]
Percentage of waveforms without the second systolic peak	%no_sys2	Percentage of 10-s intervals in which no sys2 was detected.	Schaafsma A. Improved parameterization of the transcranial Doppler signal. Ultrasound Med Biol 2012;38:1451–1459.	De Goede 2017 [42]
Transient hyperemia response ratio or Transient hyperemia response test	THRR (Feng 2021) or THRT (Crippa 2022)	CBF is analyzed before, during, and after the ipsilateral compression of the carotid artery at the neck level. Flow must undergo a reduction of 30–50% from baseline to ensure a proper compression. Compression duration is between 3 and 9 s. After the occlusion is released, blood flow rapidly increases (hyperemia), and velocity is usually higher than the baseline due to a vasodilation occurring during compression. The ratio between maximal post-release (five heartbeats) and baseline PSV is measured. A THRR index above 1.09 (>10% increase) is regarded as indicating dynamic cerebral vascular autoregulation function; if the level falls below 1.09, this is regarded as indicating impairment of CAR.	Cavill et al. Factors affecting assessment of cerebral autoregulation using the transient hyperaemic response test. Br J Anaesth. (1998) 81:317–21 [68]	Feng 2021 [24]
			Zeiler et al., Pressure Autoregulation Measurement Techniques in Adult Traumatic Brain Injury, Part I: A Scoping Review of Intermittent/Semi-Intermittent Methods. J. Neurotrauma 2017, 34, 3207–3223. [69]	Crippa 2022 [34]
Gain, phase, coherence	-	Gain phase and coherence are transfer function analysis metrics that compare two signals in their spectrum frequency (ABP and MCAv). They quantify the effectiveness of dynamic CAR as a filter that dampens MAP-induced changes in CBF. In particular, gain compares the amplitude of the signals hypothesizing that high amplitude oscillations in ABP should be dampened in CBF. Phase refers to the displacement of the CBF signal relative to the MAP signal, which reflects the response time of dynamic CAR. Coherence quantifies the linearity between the spectral power of CBF and the spectral power of MAP, assuming that when signals are highly related, changes in ABP are passively transmitted to CBF and CAR is impaired.	Zhang R, Zuckerman JH, Giller CA, Levine BD. Transfer function analysis of dynamic cerebral autoregulation in humans. Am J Physiol 1998;274:H233–41. [70] Panerai RB, Dawson SL, Potter JF. Linear and nonlinear analysis of human dynamic cerebral autoregulation. Am J Physiol 1999;277:H1089–99. [71] Meel-van den Abeelen AS, van Beek AH, Slump CH, Panerai RB, Claassen JA. Transfer function analysis for the assessment of cerebral autoregulation using spontaneous oscillations in blood pressure and cerebral blood flow. Med Eng Phys 2014;36:563–75. [72]	Berg 2015 [67], Berg and Plovsing 2016 [22]
Optic nerve sheet diameter	ONSD		Wang, L.J.; et al. Non-invasive and quantitative intracranial pressure estimation using ultrasonographic measurement of optic nerve sheath diameter. Sci. Rep.2017, 7, 42063 [73]	Czempik 2020 [46]

**Table 7:** The indexes used for autoregulation estimation in the body of evidence analyzed. To note, this is not the complete list of all the ways to estimate autoregulation.

	Dynamic AR	Static AR
**Snapshot metrics, qualitative**	THRT—Transient hyperemia response test	-
**Prolonged monitoring required, quantitative**	ARI—Autoregulation index Mxa—Mean flow index (assessed between ABP and MCAv) Transfer function analysis indexes (phase, gain, coherence)	CAI—Cerebral autoregulation index IOR—Index of autoregulation

## Supplementary Materials

The following supporting information can be downloaded at: https://www.mdpi.com/article/10.3390/jcm13226920/s1, Attachment S1: Search strategy; Attachment S2: Data extraction summary table; Attachment S3: Sepsis definitions over time.

## Abbreviations

ABP	arterial blood pressure
APACHE	Acute Physiology and Chronic Health Evaluation
ARI	autoregulation index
BBB	blood brain barrier
CAR	cerebral autoregulation
CBF	cerebral blood flow
CPP	cerebral perfusion pressure
eCPP	estimated cerebral perfusion pressure
EEG	electro encephalogram
ICP	intracranial pressure
ICU	intensive care unit
MAP	mean arterial pressure
Mx and Mxa	mean flow index
nICP	non-invasive intracranial pressure
PI	pulsatility index
POCUS	point of care ultrasound
RI	resistance index
SAD	sepsis-associated delirium
SAE	sepsis-associated encephalopathy
TCD	transcranial doppler
TCCS	transcranial color-coded sonography
THRT	transient hyperemia response test
