# Supplementary material for: Brain Ultrasonography in Critically Ill Septic Patients: A Scoping Review
Source: J Clin Med. 2024 Nov 17;13(22):6920. doi: 10.3390/jcm13226920 (PMC11595057; doi:10.3390/jcm13226920)
Supplement: Supplementary file 1 [file jcm-13-06920-s001.zip › Attachment 1 - search strategy.pdf]

Search strategy by database

Pubmed:

- ("ultrasonography, doppler, transcranial"[MeSH Terms] OR "ultrasonography, doppler, transcranial"[MeSH Terms] OR "transcranial doppler"[All Fields] OR "transcranial sonography"[All Fields] OR "transcranial ultrasound"[All Fields] OR "transcranial color doppler"[All Fields] OR "TCCD"[All Fields] OR "TCCS"[All Fields] OR "transcranial color coded sonography"[All Fields] OR "transcranial color coded doppler"[All Fields]) AND ("sepsis"[MeSH Terms] OR "sepsis"[Title/Abstract] OR "septic shock"[Title/Abstract] OR "septic encephalopathy"[Title/Abstract] OR "shock, septic"[MeSH Terms] OR "sepsis associated encephalopathy"[Title/Abstract])

Scopus:

- (INDEXTERMS("doppler transcranial ultrasonography") OR INDEXTERMS("doppler sonography, transcranial") OR "transcranial doppler" OR "transcranial sonography" OR "transcranial ultrasound" OR "transcranial color doppler" OR TCCD OR "transcranial color coded sonography" OR "transcranial color coded doppler") AND (INDEXTERMS(sepsis) OR TITLE-ABS(sepsis) OR TITLE-ABS("septic shock") OR TITLE-ABS("septic encephalopathy") OR TITLE-ABS("sepsis associated encephalopathy") OR INDEXTERMS("septic shock"))

Web of Science:

- (TS=(sepsis) OR TS=(septic shock) OR TS=(sepsis associated encephalopathy) OR TS=(septic encephalopathy)) AND (ALL=(doppler transcranial ultrasonography) OR TS=(transcranial doppler) OR TS=(transcranial sonography) OR TS=(transcranial ultrasound) OR TS=(TCCD) OR TS=(TCCS) OR TS=(transcranial colour-coded doppler) OR TS=(doppler transcranial sonography) OR TS=(transcranial colour-coded sonography))
