# Supplementary material for: Brain Ultrasonography in Critically Ill Septic Patients: A Scoping Review
Source: J Clin Med. 2024 Nov 17;13(22):6920. doi: 10.3390/jcm13226920 (PMC11595057; doi:10.3390/jcm13226920)
Supplement: Supplementary file 1 [file jcm-13-06920-s001.zip › Attachment 2 - Data extraction summary table.pdf]

Table S1. Data extraction summary table. Abbreviations: ARI: autoregulation index. CAI: cerebral autoregulation index. CAR: cerebral autoregulation. CBF: cerebral blood flow. CBFi: cerebral blood flow index. CCP: capillary closing pressure. CCT: cerebral circulation time. CD: cognitive decline. $CMRO_2$: cerebral metabolic rate of oxygen. CO_2R: CO_2 reactivity. CPP: cerebral perfusion pressure. CRC: cerebrovascular reserve capacity. CVR: cerebrovascular resistances (unless otherwise specified). eCBF: estimated CBF. eCPP: estimated cerebral perfusion pressure. EDV: end diastolic velocity. ICA: internal carotid artery. ICP: intracranial pressure. ICU: intensive care unit. IFV: intravascular flow volume. IOR: index of autoregulation. MAP: mean arterial pressure. MCA: middle cerebral artery. Mx or Mxa: mean flow index. NCR: normalised CO_2 reactivity. nICP: non-invasive intracranial pressure. ONSD: optic nerve sheet diameter. PI: pulsatility index. PSV: peak systolic velocity. RI: resistance index. SABD: sepsis associated brain dysfunction. SAD: sepsis associated delirium. THRR: transient hyperemia response ratio. THRT: transient hyperemia response test.

Author and year	Type of study	Sepsis definition used	Population and Context	Age of the septic patients median [IQR] or median (range) or mean ± SD	TCD metrics	Objectives	Intervention	Results	Time of observation	Conclusions
Matta 1996	Observational, Cohort	Bone et al (*1989)*	10 patients with sepsis syndrome and altered mental state<br><br>ICU; sedated and ventilated	60 (21–86)	MCAv; IOR, CO_2R<br><br>Snapshot metrics.	To assess if CAR and CO_2R are impaired in sepsis syndrome.	Phenilephrine infusion to increase MAP by 20mmHg; hypoventilation to increase $PaCO_2$ (3 to 7kPa). 3 measures of MCAv during phenylephrine and CO_2 test (start-middle-end).	Mean IOR was 0.92 (intact autoregulation). CO_2R was within normal limits for all patients.	Within 24 h of admission	-
Straver 1996	Observational, Cohort	Bone et al (*1989)*	20 patients with septic shock.<br><br>ICU; inotropes plus vasoactive, sedatives and mechanical ventilation	60 (20-87)	MCAv; PI; MCA/ICA index<br><br>Not specified if snapshot metrics.	To assess if the severity of sepsis and its hemodynamic changes are reflected in the TCD signal	Hemodynamic monitoring (PAC) + TCD monitoring	Inverse relationship between SVRI and mean and diastolic MCAv. MCA/ICA index and MAP showed an inverse relationship. MCA and ICA flow velocities abnormalities are more pronounced in severe disease (higher APACHE II).<br>MCAv and ICA velocities were higher in non-survivors.	5 to 8 consecutive days; no reference to time from admission or onset of symptoms	Acute hyperventilation may reduce CBF. TCD seems a valuable tool to monitor potential critical brain ischemia.
Terborg 2001	Observational, Cohort	Bone et al (*1992)*	8 patients with severe sepsis or septic shock.<br><br>ICU ventilated patients with inotropic +/- vasopressor support.	59 [56-63.5] median IQR	MCAv, NCR<br><br>Snapshot measure.	To evaluate if CO_2R is impaired in patients with severe sepsis or septic shock.	TCD and NIRS monitoring during hypercapnia induction through hypoventilation (ventilated patients) or CO_2 breathing (spontaneously breathing patients).	During septic shock, NCR was significantly reduced. MCAv corrected for baseline $EtCO_2$ during normocapnia did not differ during and outside sepsis.	During and outside an episode of severe sepsis or septic shock.	Our study confirms NIRS as an additional method for testing a reduced CO_2R in sepsis. NIRS reactivities were significantly related to NCR.
Bowie 2003	Observational, Cohort	Bone et al (*1992)*	12 patients with sepsis.<br><br>ICU sedated and ventilated; inotropes and vasoactive drugs as clinically indicated.	68 (39-83)	CO_2R<br><br>Probe fixed in place, but measure recorded as a snapshot	To study the effects sepsis on CO_2R.	Ventilation modification to achieve $EtCO_2$ +1 and -1 kPa from baseline.	Abnormal CO_2R in 10/12 patients (high variability). CO_2R didn't significantly affect the outcome (mortality) and was not related to APACHE II, hemodynamic parameters, temperature, WBC count.	After more than 24 hours of established sepsis	The cerebral vascular response to CO_2 may be significantly affected; its impact on outcome remains to be established.
Thees 2007	Observational, Cohort	Bone et al (*1992)*	10 patients with sepsis showing EEG abnormalities. 10 healthy controls only for EEG recordings.	44 (3-74)	CO_2R, CCP; CBF, $CMRO_2$.<br><br>Continuous measurements (minutes)	To determine if CBF and oxygenation alterations may play a role in SAE	Hyperventilation to decrease $EtCO_2$ of 10mmHg	Decrease in CBF and MCAv were observed during hyperventilation (21.8±4.8%/kPa). Mean SjO2 decreased (75±8% to 67±14%). CCP increased (25±11 to	Sepsis for more than 48 hours	None of the recorded parameters of cerebral perfusion and oxygenation seemed causative for SAE (EEG or clinical

			ICU, mechanically ventilated.					39±15mmHg). CO_2R was not impaired. All the survivors (8/10) showed a pathologic neurological examination during the first 5 days after extubation.		neurological exploration).
Pfister 2008(2)	Observational, Cohort	Levy et al (2003)	16 patients with sepsis or severe sepsis or septic shock.<br><br>ICU. Some intubated and some not.	74.5 (18 to 90)	MCAv, Mx<br><br>Continuous, 1hr recording	To test if: (1) CBF is altered in SAD; (2) IL-6 and CRP are associated with SAD; (3) s100β and cortisol are markers for SAD.	1hr TCD monitoring plus CRP, IL-6, cortisol dosage; CAM-ICU scale	12/16 patients presented SAD. No differences in CBF between SAD and non-SAD groups. Mx was altered in SAD patients (impaired CAR). CRP was higher.	Within 48 hrs of admission to the ICU, after stabilization.	Cerebral perfusion did not differ between patients with and without SAD. The state of CAR differed significantly between the two groups.
Pfister 2008	Observational, Cohort	Levy et al (2003)	16 patients with sepsis, severe sepsis or septic shock.<br><br>ICU. Some intubated and some not.	67±17	MCAv, nICP, eCPP<br><br>Continuous, 1hr recording/daily	To test if aggressive fluid administration could lead to cerebral edema, ICP increase, and CPP reduction.	1hr TCD monitoring plus CRP, IL-6, cortisol dosage; CAM-ICU scale. Daily investigations up to four consecutive days (45 measures/15 patients).	nICP>15 mmHg was measured on at least 1 day in 47% of patients. The nICP increases were moderate and never exceeded 20 mmHg. nICP correlated strongly with MAP ($r=0.63$, $p<0.0001$). Patients who died did not have higher peak nICP than patients who survived: (16±3 vs 16±3 mmHg, $p=0.89$). Reductions in eCPP on at least 1 day were found in 73% (CPP<60mmHg) and 20% (CPP<50mmHg) of patients. Low eCPP correlated significantly with high S-100β levels ($r=-0.47$, $p=0.001$). No significant correlations between nICP and overall or daily fluid administration or balance.	Not specified	Moderate elevations in nICP seem to occur in patients with sepsis, independently of the amount of fluid administered. As MAP levels are typically low in sepsis, even small increases in ICP may negatively affect CPP.
Steiner 2009	Observational	Not specified	23 patients with sepsis, severe sepsis or septic shock.<br><br>Surgical ICU	68 (mean)	Mx<br><br>Patients were monitored for 1 h every day on up to four consecutive days.	To compare a validated TCD index of autoregulation (Mx) with a NIRS measure of autoregulation (Tox) in a group of patients suffering from sepsis, severe sepsis, or septic shock.	1hr TCD monitoring/daily for up to 4 consecutive days. Total of 81 recordings for 23 patients.	Coherence analysis indicated a good level of association between the two trends. The regression between Tox and Mx showed a strong positive association ($R = 0.81$; $P < 0.0001$). $PaCO_2$-induced dilatation of flow-regulating vessels was associated with worse autoregulation.	Not specified	Waves of periods 20s-2min can be recorded in Tox as well as Mx. TOI and Mx are in agreement in adults with sepsis.
Taccone 2010	Clinical Trial	Levy et al (2003)	21 patients with septic shock.<br><br>ICU. Mechanically ventilated.	65 (43-81)	CAI, CVR (cerebrovascular reactivity), MCAv.<br><br>Continuous during the	To evaluate CAR and assess the influence of $PaCO_2$ on CAR capacity in patients with septic shock.	Increase of MAP with norepinephrine to achieve 4 different steps of MAP.	Autoregulation was impaired in 66% of patients; the % of patients with impaired autoregulation increased for increasing values of $PaCO_2$.	Septic shock for less than 72h.	CAR is impaired in most patients with septic shock. $PaCO_2$ concentrations above 40 mmHg are more likely to alter CAR.

					manoeuvre of MAP increase.					
Szatmári 2010	Observational, Case Control	Bone et al (*1992)*	14 patients with sepsis presenting SAE + 20 healthy controls.<br><br>ICU, without hemodynamic compromise, not ventilated, no organ failure	Not reported	MCAv, PI, CVR (cerebrovascular reactivity), CRC.<br><br>Snapshot. Baseline plus 20min recording during acetazolamide test	To test whether acetazolamide-induced cerebral VMR is altered in patients with SAE	15mg/Kg acetazolamide infusion and 20min monitoring with TCD.	Resting mean and the diastolic blood flow velocities were lower in the group with sepsis. PI was higher. Vasomotor response was slower and lower in sepsis (less CRC, lower systolic MCAv).	Not specified	CAR and metabolic regulation occur at the same level (resistance arterioles). The ability of resistance arterioles to dilate was decreased in SAE.
Fülesdi 2012	Observational, Case Control	Bone et al (*1992)*, Levy et al (2003)	16 patients with severe sepsis and at least 2 organ dysfunctions + 16 healthy controls.<br><br>ICU, 14/16 mechanically ventilated	74 (37 - 88)	MCAv, PI, CVR, CRC.<br><br>Snapshot. Baseline plus 20min recording during acetazolamide test	To test whether acetazolamide-induced cerebral VMR is altered in patients with SAE	15mg/Kg acetazolamide infusion and 20min monitoring with TCD.	PI was higher in septic patients. MCAv didn't differ. CRC was similar in the two groups while CVR decreased slower in the septic group (more prolonged vasodilatory response).	Within 24 hours after diagnosing severe sepsis	In early sepsis the ability of the brain resistance arterioles to dilate is decreased.
Schramm 2012	Observational, Cohort	Levy et al (2003)	30 patients with severe sepsis (5) or septic shock (25).<br><br>ICU, sedated and ventilated.	64 ± 17 years (mean SD)	Mx<br><br>1hr prolonged recording. Daily measures up to four days.	To study if CAR is impaired in early sepsis and if impairment is correlated with SAD	1hr daily TCD monitoring for 4 days; CRP, PCT, NSE and s100β dosage. CAM-ICU as SAD screening. EEG in case of delirium.	25 patients (88%) showed impaired CAR during the four days with a decreasing prevalence during days (day1 - 60%, day2 - 59%, day3 - 41%, day4 - 46%). SAD developed in 76% of patients and was not related to EEG. The status of CAR at day 1 was related to SAD development at day 4. SAD was associated with age.	Within 24 hours after the first signs of sepsis became obvious.	CAR is impaired in the majority of patients with severe sepsis and septic shock during the first 2 days. SAD was frequently diagnosed (76% of patients). CAR impairment was associated with SAD, suggesting that CAR might be a contributing factor.
Pierrakos 2013	Observational, Case Control	Levy et al (2003)	20 septic patients + 21 non septic patients (controls).<br><br>ICU	67 ± 11 (mean SD)	MCAv, PI, RI, eCBF<br><br>Two snapshot measures.	To evaluate the feasibility and efficacy of TCD in assessing cerebral perfusion changes in septic patients. To assess static cerebral perfusion characteristics and changes in septic versus non-septic critically ill patients.	Two TCD measures in 2 days, with at least 20hrs between them. Each measurement derived from the mean of three snapshot measures on both sides.	TCD has a feasibility of 91% vs. 85%, p = 0.89 (septic vs controls) due to acoustic bone window. PI and RI were higher in patients with sepsis than controls and higher in the first day.	Within 48 hrs after confirmation of sepsis diagnosis.	Cerebral vascular constriction is detectable by TCD in the early stage of sepsis. TCD can be a useful tool to evaluate cerebral vascular tone and possibly cerebral perfusion in critically ill septic patients.
Toksvang 2014	Observational, Cohort	Levy et al (2003)	8 patients with severe sepsis or septic shock. 6 patients' data had been already used in a previous study (Berg et al 2012).	62 ± 11 (mean SD)	MCAv.<br><br>Snapshot measure. 15 (11-22) min recordings.	To compare TCD and NIRS for the bedside assessment of changes in CBF in patients with severe	Progressive MAP increase (25-30mmHg) using noradrenaline. CBF plus ABP and NIRS recording during the MAP challenge. 1 recording	The MAP increase generated a mean increase in MCAv of 14% (2-22%). There was poor agreement between TCD	Severe sepsis or septic shock diagnosis within the past 72 hours	TCD and spatially resolved NIRS cannot be used interchangeably for monitoring and evaluating the

			ICU ventilated patients. 5 with shock and vasopressor support.			sepsis or septic shock.	daily up to three consecutive days (8 patients/15 recordings).	and NIRS for CBF estimation.		cerebral haemodynamic impact of noradrenaline treatment in critically ill patients with sepsis.
Pierrakos 2014	Observational, Cohort	Levy et al (2003); Septic shock was defined as the need of noradrenaline support of more than 0.1 μg/kg/min.	40 patients with sepsis or septic shock.<br><br>ICU.	Age reported by group: PI<1.3: 62 ± 16. PI>1.3: 72 ± 13.	MCAv, PI, CBFi.<br><br>Two snapshot measures (10 seconds monitoring): (1) on the first 24 hours from sepsis diagnosis and (2) three days later.	To correlate the clinical presentation of SAE (delirium) with changes in PI, as evaluated by TCD	TCD monitoring (10 seconds image acquisition) at day 1 and after three days. CAM-ICU at day one and day three.	Twenty-one patients (55%) presented delirium. PI on the first day was a good predictor of the presence of confusion (AUC = 0.908, 95%, CI 0.80-0.98, p < 0.01). For a cut-off value of 1.3, there was a 95% sensitivity and an 88% specificity. PI was related to confusion independently from age or APACHE II score (multivariate regression analysis).	Sepsis for fewer than 24 hours.	Cerebral vascular constriction detected by TCD in the early stages of sepsis, is correlated with clinical signs of SAE. A cut-off value of PI > 1.3 could be used in clinical practice as a risk factor for delirium in septic patients. The evolution of cerebral perfusion could vary between patients during sepsis.
Berg 2015	Observational, Cohort	Not specified	10 patients with severe sepsis or septic shock. Part of the patients' data had already been used for Toksvang et al, 2014, and Berg et al, 2012.<br><br>ICU	64 (45-74)	MCAv; gain, phase and coherence between ABP and CBF; data analyzed in the very low (0.02–0.07 Hz), low (0.07–0.20 Hz) and high (0.20–0.30 Hz) frequency ranges<br><br>Prolonged (15-20min)	To compare NIRS- to TCD-based estimates of dynamic CAR.	Single 15-20min steady-state recording of simultaneous invasive ABP, NIRS and TCD signals. 3 patients had two recordings over two days, and 1 patient had three recordings over three days.	There were no significant correlations between NIRS and TCD-based estimates in any of the frequency ranges (poor agreement).	Severe sepsis or septic shock within the past 72 h.	A CAR impairment in patients with sepsis (determined by TCD) may remain undetected by NIRS.
Berg 2016	Observational, Cohort	Levy et al (2003)	16 patients presenting severe sepsis (11) or septic shock (5). All patients were analysed for baseline metrics. Only 7 patients underwent the intervention and thus were further analysed. Part of the patients' data had already been used for Berg et al 2015, Toksvang et al, 2014, and Berg et al, 2012.<br><br>ICU mechanically ventilated.	Age reported only for the 7 patients that underwent intervention: 58 (43-74).	MCAv, CVR; gain, phase and coherence between ABP and CBF; data were analyzed in the very low (0.02–0.07 Hz), low (0.07–0.20 Hz) and high (0.20–0.30 Hz) frequency ranges.<br><br>Prolonged (20min)	To investigate if reducing $PaCO_2$ by short-term mechanical hyperventilation would cause cerebral vasoconstriction and enhance dynamic CAR in patients with severe sepsis or septic shock.	Hyperventilation with a target of 10-20% $EtCO_2$ reduction (↑ in minute ventilation) in 7/16 patients.	Hyperventilation was associated with an increase in gain in the low frequency range. Hyperventilation was associated with a 36% (18–48) increase in CVR (1.3 [1.1–2.7] to 2.5 [2.1–3.9] mmHg sec/cm, p<0.05), and a consequent 22% (11–37%) reduction in MCAv (57 [33–68] to 32 [21–40] cm/sec, p<0.05). This yielded a 30% (27–66%) change in MCAv per kPa of $PaCO_2$.<br>At baseline phase was inversely and significantly	Severe sepsis or septic shock diagnosis within the past 72h.	CO_2R is preserved in critically ill patients with sepsis. Short term mechanical hyperventilation does, however, not necessarily enhance CAR.

								correlated with $PaCO_2$ (ρ=-0.87; p<0.01).		
Pierrakos 2016	Observational, Cohort	Calandra et al (2005)	28 patients with sepsis or septic shock.<br><br>ICU	71 (38-89)	MCAv, PI, CBFi.<br><br>Snapshot daily measures, but time and duration not specified.	To assess the relationship between cerebral perfusion abnormalities on the onset of sepsis and possible CD after sepsis resolution.	TCD monitoring on day 1 and 3 of sepsis. CAM-ICU during the ICU stay and at discharge, plus MMSE if CAM-ICU negative, or clock drawing test if MMSE not valid (<12 years of schooling).	50% of patients presented CD at the time of discharge. Differences in TCD parameters between the two groups were found only on the first day of the study. PI was higher in patients with CD (2.2 ± 0.7 vs. 1.4 ± 0.5, p = 0.02) and CBFi was lower (363±170 vs. 499±133, p = 0.03). In univariate analysis, delirium and PI on the first day of the study were related to CD (OR: 36.1, 95%CI 4.3–299.1, p = 0.01, OR:4.1, 95%CI 1.1–15.2, p = 0.03). In the multivariate analysis PI was not found to be related to CD independently of the presence of delirium.	On the first and third days after sepsis onset.	Microcirculation abnormalities are not related with CD after sepsis resolution in the absence of severely decreased CBF. Delirium is an independent risk factor for cognitive impairment in sepsis patients who were discharged from ICU.
De Goede 2017	Observational, Case Control	Bone et al *(1992)*, Levy et al (2003)	16 patients with severe sepsis or septic shock + 42 controls (from normal tilt-table tests).<br><br>ICU with vasopressor support and mechanically ventilated.	70 (mean), 44-88 (range)	acc, sys1, sys2, dias@560, %no_sys2<br><br>Snapshot before and after fluid challenges.	To investigate whether an alternative set of TCD parameters can identify changes in cerebral hemodynamics after adequate fluid resuscitation in critically ill septic patients.	TCD monitoring for at least 1 minute before and after each fluid challenge (boluses of 500ml in accordance with clinical needs and intensivist choice). After recording, 10 beats were used to calculate the metrics of interest with a software developed for the scope.	Patients with sepsis presented sys2 in only 2 of 16 cases (sys2 was present in all the control group patients). The presence of sys2 increased during resuscitation (decrease in %no_sys2, p<0.019). Acc, sys1 and sys2 increased during resuscitation, as well as systolic ABP (p=0.048, p=0.022, p=0.005 and p=0.031, respectively).	Before fluid resuscitation was started, when possible, otherwise during/after resuscitation was started	Adequate fluid resuscitation in patients with sepsis significantly increased the systolic but not the diastolic components of the MCAv. Most septic patients showed sys2 absence that recurred and increased during the period of fluid resuscitation. Reappearance of sys2 may be used as an alternative criterion for hemodynamic monitoring. Repetitive TCD measurements during resuscitation are feasible.
Le Dorze 2018	Observational, Case Control	Not reported	38 patients with severe sepsis and septic shock + 17 controls (patients undergoing anesthesia for orthopedic surgery) + 11 brain injured patients (TBI or stroke).<br><br>OR plus ICU. All patients receiving sedatives (propofol) and under mechanical ventilation.	64 [54–79] median, IQR	MCA PSV and end EDV blood flow velocities.<br><br>Snapshot with handheld probe.	To investigate modifications of CBF induced by a fluid challenge increasing CO and SV; to evaluate the impact of systemic inflammation on the CO/CBF relationship; to compare results with patient having brain injury but no	Acquisition of TCD plus trans-esophageal parameters before and after a fluid challenge.	At baseline: hemodynamic parameters were different in the three groups (Baseline CO and HR higher, MAP lower in the sepsis group). PSV was higher in the sepsis group than in the control group but not with BI group.<br>After the fluid challenge: PSV and EDV increased	Reported 0-3 days from admission	The increase in SV (and CO) after a fluid challenge increased PSV and EDV only in patients exhibiting severe acute systemic inflammation. The impact of a systemic blood flow increase predominated the role of MAP rise on CBF.

						detectable systemic inflammation.		significantly (p<0.0042, p=0.0001) only in the sepsis group; EDV increased significantly in the sepsis group in relation to the BI group. No significant correlations between systemic and cerebral hemodynamic changes were observed in any group.		
Crippa 2018	Observational, Cohort	Levy et al (2003)	100 patients with severe sepsis or septic shock.<br><br>ICU.	63 [52–72] median [IQR]	Mxa.<br><br>Prolonged for Mxa assessment. The length of recording was 13 [10–18] minutes per patient. One snapshot measure.	To evaluate the association of altered CAR with the occurrence of SABD, as well as to identify clinical factors associated with altered CAR in patients with sepsis.	TCD monitoring for at least 10 minutes	Mxa was 0.29 [0.05–0.62], and 50% of patients had impaired CAR. In the multiple linear regression analysis, lower MAP, history of CKD, and fungal infections were independently associated with higher Mxa. There was no difference in Mxa between survivors and non-survivors at ICU discharge. SABD was more common in patients with altered CAR (34 of 50 [68%] vs 23 of 50 [46%]; p=0.04), and Mxa was higher in patients with SABD (0.47 [0.21–0.64] vs 0.23 [-0.12–0.52]; p <0.01). In multivariable analysis, higher Mxa, vascular disease and mechanical ventilation were independent predictors of SABD. The best Mxa cut-off to predict SABD was 0.18 (sensitivity 79%, specificity 47%).	Within 48hours from sepsis or septic shock diagnosis.	CAR assessed by Mxa was altered in half of the cases. Altered CAR was found to be independently associated with SABD, but not with survival at ICU discharge.
Czempik 2020	Observational, Cohort	Singer et al (2016)	10 septic shock patients.<br><br>ICU. Sedated and ventilated patients	65 (50–78) (median, IQR)	ONSD<br><br>Snapshot.	To analyze ONSD in septic shock patients and its potential role in screening for SABD. To investigate the correlation between ONSD and sepsis severity scores.	ONSD bilateral measurement every 24 hours for up to 10 consecutive days (n = 1) until discharge (n = 3) or death (n = 6).	49/80 ONSD measurements performed (61%) exceeded 5.7mm. No correlation between ONSDs and CRP concentrations, highest daily lactate, or SOFA.	Not specified.	ONSD above the cut-off value is common in septic shock patients and fluctuates around upper limit. ONSD measurement should be applied for screening of SABD cautiously.
Feng 2021	Observational, Cohort	Singer et al (2016)	51 septic shock patients.<br><br>ICU	53±11 (mean, SD)	MCAv, CBFi, PI, THRR.<br><br>Snapshot.	To identify variations in the cerebral hemodynamic indices of the MCA or in cerebral/peripheral StO_2 that increase the risk of	TCD measures, biomarkers, continuous brain oxygen saturation (rSO_2), continuous thenar eminence saturation (StO_2), CAM-ICU, echocardiography.	The SAD group exhibited lower levels of diastolic MCAv (49.7±20.3 vs. 61.9±17.3 cm/s, p=0.026) with a higher PI (0.98±0.19 vs. 0.84±0.20, p=0.019). The SAD group had a significantly higher	TCD: once 6 h of initial resuscitation had been undertaken. NIRS: continuous recording from	Patients with SAD have a close correlation with poor outcomes. Independent risk factors for SAD were mean rSO_2 <55% and cerebrovascular

						developing SAD. Primary outcomes: discharge from ICU or development of delirium within 7 days of ICU admission; secondary outcome: mortality rate after 28 days.		level of cerebrovascular dysfunction (THRR index < 1.09, 40 vs. 10%, p=0.01). The logistic regression analysis demonstrated that several independent risks were SAD predictors: rSO_2 <55% [OR=3.864, 95% CI: 1.026 to 14.550, p=0.046] and the THRR index<1.09 (OR=5.77, 95% CI: 1.222–27.255, p=0.027).	several 60 mins once the septic shock patients had 6h of initial resuscitation. Biomarkers: not specified. Echocardiograp hy: within 60min before ICU admission.	dysregulation (THRR <1.09).
Crippa 2022	Observational, Cohort	Singer et al (2016)	40 patients with sepsis.<br><br>ICU	74 [63–79] median [IQR]	THRT, eCPP, eICP.<br><br>Snapshot.	To assess the correlation between PLR-derived variables with the assessment of CAR and non-invasive eCPP and eICP in critically ill patients with sepsis.	Pupillometry plus THRT test once after admission.	53% patients had impaired CA. 55% patients had low eCPP; median eICP was 9 [4–24] mmHg and 38% of patients had high eICP. 35% of patients had low eCPP and high eICP; 20% of patients had low eCPP and normal eICP. 45% of patients had normal eCPP and either high eICP (2%) or normal eICP (43%). Dilation velocity was significantly lower in patients with impaired CAR (1.3 [1.2–1.9] vs. 2.6 [1.8–3.2] mm/s; p < 0.01). The AUROC for dilation velocity to predict impaired CAR was 0.78 [95% CI 0.63–0.94], with a value of <2.2 mm/s having a sensitivity of 85% [95% CI 65–95] and a specificity of 69% [95% CI 46–84]. NPi values were lower in patients with low compared to normal eCPP (3.7 [3.5–4.1] vs. 4.6 [4.5–4.6], p < 0.01) and in patients with high eICP compared to normal eICP (3.5 [3.5–3.6] vs. 4.5 [4.3–4.6]; p < 0.01). NPi was correlated with eCPP (r = 0.77, p < 0.01) and eICP (r = -0.87, p < 0.01).	Within 48 hours of ICU admission	Pupillary dilation velocity was lower in patients with impaired CAR. Lower NPi values were observed in patients with low eCPP and high eICP. Automated pupillometry may play a role in assessing brain hemodynamics in septic patients.
Caldas 2022	Observational, Cohort	Singer et al (2016)	95 patients with sepsis.<br><br>ICU.	64 (±13)	ARI, Mxa.<br><br>Snapshot	To compare the assessment of CAR using ARI and Mxa in a large cohort of patients with sepsis.	TCD monitoring sessions to calculate indexes of autoregulation.	Median ARI and Mxa values were 4.38 [2.83–6.04] and 0.32 [0.14–0.59], respectively; there was no significant correlation between ARI and Mxa (r=−0.08; p=0.39). Impaired CAR according to the ARI	Within 72 hours from sepsis diagnosis	Mx and ARI, two common indexes of CAR estimation, had a weak correlation and a poor agreement to classify CAR. These findings underline the limitations in comparing results

								threshold was observed in 42% of patients; impaired CAR according to Mxa threshold was observed in 53% patients. ARI and Mxa were concordant in classifying 24% of patients with impaired CAR and 29% of patients with intact CAR; a poor agreement between the two indices to categorize CAR was therefore obtained (Cohen's kappa coefficient = 0.08).		from different studies about CAR. A standardization for the CAR assessment is warranted.
Crippa 2022	Observational, Cohort	Not specified	15 patients with sepsis.<br><br>ICU, ventilated.	57 [41–70]	MCAv, Mxa.<br><br>Snapshot: monitoring sessions of 11 [10-13] min.	To investigate whether modifications in $PaCO_2$ would affect CBF modifications in response to spontaneous fluctuation of MAP in septic patients.	TCD monitoring sessions during ventilation modifications to achieve a low or high CO_2 target. TCD to calculate indexes of autoregulation.	MCAv was lower at low CO_2 compared with high CO_2 (57 [43–78] vs. 74 [55–88] cm/s, p<0.01). There were no differences in Mxa between low CO_2 and high CO_2 (0.35 [0.27–0.52] vs. 0.47 [0.02–0.77], p=0.76).	Within 48 hours from sepsis diagnosis	Moving from lower to higher $PaCO_2$ values resulted in a significant increase in CBF velocities. $PaCO_2$ changes did not significantly affect dynamic CAR in critically ill patients with sepsis.
Zheng 2023	Observational, Cohort	Singer et al (2016)	198 patients with sepsis (65 with SAE and 133 non-SAE).<br><br>ICU	SAE group: 70 [57–78] non-SAE group: 67 [59–77]	MCAv, PSV, EDV, PI, RI, IFV, CBF volume.<br><br>Snapshot.	To observe the CBF changes in the early stage of sepsis and explore its relationship with the incidence of SAE.	TCD measurements plus data collection of various clinical (GCS plus CAM-ICU) and biomarkers parameters (NSE, CRP, PCT, cytokines).	The SAE group showed higher APACHE II score (19 [14–22] vs 15 [12–19], P=.001), serum NSE level (17.8 [12.7–28.6] ng/mL vs 12.3 [9.1–16.3] ng/mL, P<.001), and hospital mortality (36.9% vs 13.5%, P<.001) compared with the non-SAE group. SAE patients showed significantly elevated PSV (107 [69–138] cm/s vs 85 [69–101] cm/s, P=.002) and mean MCAv (57 [37–93] vs 54 [42–66], P=.045) only in the left MCA. The PI and RI of bilateral MCAs were significantly higher in the SAE group than in the non-SAE group (even if the values were within the normal range). Patients with agitation had higher MCAv and lower PI and RI than patients with decreased consciousness, suggesting lower CVR.	Within 24 hours of ICU admission	The present study confirmed the abnormal CBF in patients with SAE and found that different types of cerebral perfusion alterations were related to different clinical features of SAE. TCD measurements of PSV and PI might show some predictive value for the incidence of the different types of SAE.
Crippa 2024	Observational, Cohort	Singer et al (2016)	132 patients with sepsis.<br><br>ICU.	64 [52-71] median [IQR]	eCPP, eICP, Mxa.<br><br>Snapshot.	To assess eICP and eCPP values in a large cohort of critically ill septic patients and	TCD recording sessions of 9 [7–12] min.	median eCPP value was 63 (IQR 58–71) mm Hg; in particular, 44 (33%) patients had low eCPP. Also, 5 (4%) patients had	TCD data collected within 48 h from diagnosis (the first TCD exam	Brain hemodynamics were frequently altered in septic patients. However, these disturbances

						describe their alterations. To assess the relationships between eICP/eCPP values and the occurrence of SAE and in-hospital mortality.		eCPP < 50 mm Hg, and 36 (27%) patients had eCPP > 70 mm Hg. The median eICP value was 8 (IQR 4–13) mm Hg; five (4%) patients had high eICP. 86 (65%) patients had normal eCPP and normal eICP, 41 (31%) patients had low eCPP and normal eICP, three (2%) patients had low eCPP and high eICP, and two (2%) patients had normal eCPP and high eICP. SABD occurrence did not differ between patients with low eCPP and patients with normal eCPP (12 of 44 [27%] vs. 31 of 88 [35%]; p = 0.43) or between patients with high eICP and patients with normal eICP (1 of 5 [20%] vs. 42 of 127 [33%]; p = 1.00). In-hospital mortality also did not differ between patients with low eCPP and patients with normal eCPP (14 of 44 [32%] vs. 24 of 88 [37%]; p = 0.68) or between patients with high eICP and patients with normal eICP (0 of 5 [0%] vs. 38 of 127 [30%]; p = 0.32).	in the first 48 hours were taken for analysis).	were not associated with clinically relevant outcomes.
Mei 2024	Observational, Cohort	Singer et al (2016)	67 patients with sepsis (32 with SAE, and 35 non-SAE).<br><br>ICU.	non-SAE group: 58.83 ± 10.53; SAE group: 57.44 ± 9.55	MCAv, PSV, EDV, PI, RI, CCT.<br><br>Snapshot.	To evaluate the efficacy of CCT as a predictive marker for SAE in the ICU setting. Subsequently, the study will explore the potential of CCT in optimizing the prediction of SAE occurrence, with the aid of a nomogram.	TCD recording plus data collection of various clinical and biomarkers parameters (NSE, S100B).	The SAE group displayed significantly elevated levels of NSE, S100B, PI, RI, and CCT, while EDV was lower (all P-values < 0.05). CCT emerged as the most efficacious predictor for SAE, with an AUC of 0.846. S100B, PI, and CCT were identified as the independent predictors for SAE. Utilizing the OR values as a basis for weighting, a nomogram was developed to illustrate the probabilistic incidence of SAE.	Within 24 hours from admission	This study revealed the superior predictive capability of CCT over other potential predictors in the assessment of SAE within ICU environments. To further enhance this predictive accuracy, the study introduced an innovative nomogram incorporating CCT, PI, and S100B. The model demonstrated robust discrimination, calibration, and clinical utility, offering a valuable tool for early intervention strategies for SAE.
