# Supplementary material for: Brain Ultrasonography in Critically Ill Septic Patients: A Scoping Review
Source: J Clin Med. 2024 Nov 17;13(22):6920. doi: 10.3390/jcm13226920 (PMC11595057; doi:10.3390/jcm13226920)
Supplement: Supplementary file 1 [file jcm-13-06920-s001.zip › Attachment 3 - sepsis definitions.pdf]

Table S2. Sepsis definitions over time.

Author	Criteria	N° of studies
**Bone et al 1989 (49)**	**Sepsis syndrome**: a systemic response to infection defined as: hypothermia (temperature less than 96 degrees F) or hyperthermia (greater than 101 degrees F), tachycardia (greater than 90 beat/min), tachypnea (greater than 20 breath/min), clinical evidence of an infection site and with at least one end-organ demonstrating inadequate perfusion or dysfunction expressed as poor or altered cerebral function, hypoxemia (PaO_2 <75 torr), elevated plasma lactate, or oliguria (urine output <30 ml/h or 0.5 ml/kg body weight without corrective therapy).<br>**Septic shock:** sepsis syndrome plus a SBP <90mmHg or a decrease from baseline in SBP >40mmHg)	2
**Bone et al 1992 (50)**	**Sepsis:** SIRS + a confirmed infectious process. In this clinical circumstance, the term sepsis represents the systemic inflammatory response to the presence of infection. SIRS is defined as more than one of the following clinical manifestations: (1) a body temperature greater than 38°C or less than 36°C; (2) a heart rate greater than 90 beats per minute; (3) tachypnea, manifested by a respiratory rate greater than 20 breaths per minute, or hyperventilation, as indicated by a PaCO2 of less than 32 mm Hg; and (4) an alteration in the white blood cell count, such as a count greater than 12,000/ cu mm, a count less than 4,000/cu mm, or the presence of more than 10 percent immature neutrophils ("bands"). These physiologic changes should represent an acute alteration from baseline in the absence of other known causes for such abnormalities, such as chemotherapy, induced neutropenia, and leukopenia. Severe sepsis= sepsis associated with organ dysfunction, hypoperfusion, or hypotension. Hypoperfusion and perfusion abnormalities may include, but are not limited to lactic acidosis, oliguria, or an acute alteration in mental status. **Septic shock:** sepsis-induced with hypotension despite adequate fluid resuscitation along with the presence of perfusion abnormalities that may include, but are not limited to, lactic acidosis, oliguria, or an acute alteration in mental status. Patients who are receiving inotropic or vasopressor agents may not be hypotensive at the time that perfusion abnormalities are measured.	4
**Levy et al 2003 (51)**	Sepsis: Infection, a documented or suspected, and some of the following:<br>1. General variables<br>a. Fever (core temperature 38.3°C),<br>b. Hypothermia (core temperature 36°C),<br>c. Heart rate >90/min or 2SD above the normal value for age,<br>d. Tachypnea,<br>e. Altered mental status,<br>f. Significant edema or positive fluid balance (20 mL/kg over 24 hrs),<br>g. Hyperglycemia (plasma glucose 120 mg/dL or 7.7 mmol/L) in the absence of diabetes,<br>2. Inflammatory variables<br>a. Leukocytosis (WBC count 12,000 L 1),<br>b. Leukopenia (WBC count 4000 L 1),<br>c. Normal WBC count with 10% immature forms,<br>d. Plasma C-reactive protein 2 SD above the normal value,<br>e. Plasma procalcitonin 2 SD above the normal value,<br>3. Hemodynamic variables: Arterial hypotension (SBP 90 mm Hg, MAP 70, or an SBP decrease 40 mm Hg in adults or 2SD below normal for age),<br>a. SvO2 70%b,<br>b. Cardiac index 3.5 L/min<br>4. Organ dysfunction variables<br>a. Arterial hypoxemia (PaO_2/FIO_2 <300),<br>b. Acute oliguria (urine output 0.5 mL/kg 1hr 1 or 45 mmol/L for at least 2 hrs),<br>c. Creatinine increase >0.5 mg/dL,<br>d. Coagulation abnormalities (INR 1.5 or aPTT>60"),<br>e. Ileus (absent bowel sounds),	8

	f. Thrombocytopenia (platelet count 100,000 L 1),<br>g. Hyperbilirubinemia (plasma total bilirubin 4 mg/dL or 70 mmol/L),<br>5. Tissue perfusion variables<br>a. Hyperlactatemia (1 mmol/L),<br>b. Decreased capillary refill or mottling.<br>**Severe sepsis:** sepsis complicated by organ dysfunction. **Septic shock** in adults refers to a state of acute circulatory failure characterized by persistent arterial hypotension unexplained by other causes. Hypotension is defined by a SBP<90 mm Hg (or, in children, 2SD below normal for their age), a MAP <60mmHg, or a reduction in SBP of 40 mm Hg from baseline, despite adequate volume resuscitation, in the absence of other causes for hypotension.	
**Calandra et al 2005 (52)**	Specific definitions were given for the most common infections in ICU: Pneumonias, Blood stream infections, Endocarditis, Catheter related infections, Urinary tract infections, Intra-abdominal infections and skin and soft tissues infections.	1
**Singer et al 2016 (53)**	**Sepsis** is defined as life-threatening organ dysfunction caused by a dysregulated host response to infection. Organ dysfunction can be identified as an acute change in total SOFA score ≥2 points consequent to the infection.<br>**Septic shock** is a subset of sepsis in which underlying circulatory and cellular/metabolic abnormalities are profound enough to substantially increase mortality. Patients with septic shock can be identified with a clinical construct of sepsis with persisting hypotension requiring vasopressors to maintain MAP ≥65 mmHg and having a serum lactate level >2 mmol/L despite adequate volume resuscitation.	7
**Sepsis def not specified**	-	3
